# Agents for Fluorescence-Guided Glioblastoma Surgery

**DOI:** 10.3390/pharmaceutics17050637

**Published:** 2025-05-11

**Authors:** Eleni Romeo, Andreas G. Tzakos, Timothy Crook, Nelofer Syed, Spyridon Voulgaris, George A. Alexiou

**Affiliations:** 1 Neurosurgical Institute, University of Ioannina, 45500 Ioannina, Greece; elenirome@gmail.com (E.R.); svoulgar@uoi.gr (S.V.); 2 Department of Neurosurgery, School of Medicine, University of Ioannina, 45500 Ioannina, Greece; 3 Section of Organic Chemistry and Biochemistry, Department of Chemistry, University of Ioannina, 45500 Ioannina, Greece; atzakos@uoi.gr; 4 Department of Brain Sciences, Hammersmith Hospital, Imperial College London, London W12 0NN, UK; tr.crook@gmail.com (T.C.); n.syed@imperial.ac.uk (N.S.)

**Keywords:** glioblastoma, 5-ALA, fluorescein, indocyanine green, fluorescence dyes, fluorophores, luminescent agents

## Abstract

Glioblastoma (GBM) is the most aggressive primary brain tumor, characterized by rapid progression and a median survival of no more than 12–18 months. Fluorescence-guided surgery is crucial, as it allows for tumor visualization and aids in its complete removal, which is essential for improving survival rates. We conducted a literature review to identify fluorescent agents that have been utilized in the removal of GBM and to assess their benefits in achieving maximum tumor resection. Our analysis focuses on their advantages, limitations, and potential impact on improving surgical precision and patient outcomes. We searched the PubMed database for studies published on fluorescence-guided resection of GBM and evaluated the utility of each agent in terms of outcomes, gross total resection (GTR), and their sensitivity and specificity for the tumor. The literature review revealed that the three agents successfully utilized are 5-aminolevulinic acid (5-ALA), sodium fluorescein, and indocyanine green. In addition to these, a variety of dyes have been investigated in studies, including peptides, lipids, and nanosystems, which appear to be very promising. To date, numerous fluorescent agents have been proposed for the surgical resection of GBM. However, 5-aminolevulinic acid (5-ALA) remains the only agent widely adopted in clinical practice, as its safety and efficacy have been well-established. Further clinical trials and studies are necessary to assess the utility, effectiveness, and potential advantages of emerging fluorescent dyes in enhancing GBM resection and improving patient outcomes.

## 1. Introduction

Globally, primary brain tumors cause 2.4% of all fatalities from cancer. Glioblastomas (GBMs, an abbreviation that was previously used for the term “Glioblastoma multiforme”) account for around half of all gliomas and are regarded as extremely uncommon, occurring in only 10 out of every 100,000 individuals worldwide [1]. However, the disease’s aggressive nature and quick progression make it a serious worldwide health concern. The median survival of patients is still only 12–18 months even with the best care, which includes maximal surgical resection, radiation therapy and temozolomide [2]. Since the tumor’s high degree of biochemical, functional, and genetic heterogeneity and its distinct tumor microenvironment allow for immune system evasion, glioblastoma cells show enhanced resistance to current therapeutic treatments [3]. Because of this, the scientific community has been working to identify novel and more effective treatments by better understanding the biology and molecular behavior of tumors and focusing on the genes and signaling pathways linked to GBM.

GBM can be considered primary or secondary depending on its clinical characteristics and degree of aggressiveness. The primary or “de novo” GBM is observed in 90% of patients diagnosed with GBM, is more aggressive, and does not exhibit precursor histological features of lower-grade malignancy. On the contrary, secondary GBM arises from low-grade or anaplastic astrocytomas, which then evolve into more aggressive forms of glioma [4]. Certain cases of mutations have been isolated that may appear and characterize each of these categories [5]. In the case of primary GBM, there are mutations in the promoter of telomerase reverse transcriptase (*TERT*), in the oncogene phosphatase and tensin homolog (*PTEN*), and overexpression of the epidermal growth factor receptor (*EGFR*). In secondary GBM, mutations are found in the *IDH1/2*, *TP53*, and *ATRX* genes [5]. In most cases of GBM, there are mutations in the p53 protein pathway and in the *RB1* gene of the retinoblastoma protein Rb with 87% and 78% of patients, respectively, according to data from The Cancer Genome Atlas Program (TCGA).

The first-line treatment for managing GBM is surgical resection. However, quite often, due to its location in vital areas of the brain, complete resection is not feasible, resulting in high recurrence rates. Recent studies have demonstrated that successfully removing at least 78% of the tumor volume is generally sufficient to improve overall survival in GBM patients. However, a further increase in median survival from 52 to 86 weeks can only be achieved through a radical resection of more than 98% of the tumor volume, highlighting the critical importance of maximizing surgical precision in GBM treatment [6].

Radiation therapy and temozolomide (TMZ) are used to target residual tumor cells after the majority of the tumor has been removed. However, this treatment alone is insufficient to prevent tumor recurrence, underscoring the need for more effective strategies to achieve maximal tumor resection [2]. To achieve the highest possible gross total resection (GTR), the scientific community has increasingly focused on developing techniques that enhance the precise delineation of GBM borders. Among these, the use of fluorescent agents has emerged as a promising approach in recent years, improving tumor visualization and aiding in more effective surgical resection.

**Figure 1:**
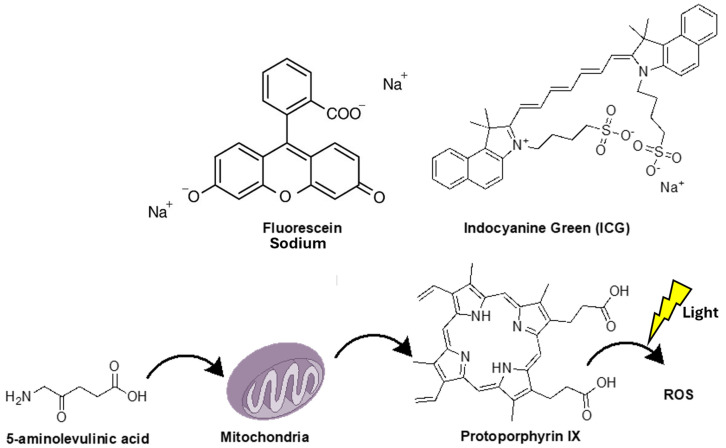
Structure of fluorescence-guided resection fluorescent agents. This figure also shows how 5-aminolevulinic acid is metabolized into protoporphyrin IX, the fluorescent dye, and how light exposure generates reactive oxygen species (ROS) during photodynamic therapy.

Fluorescence-guided tumor resection (FGR) has proven to be a highly useful, cost-effective, and efficient technique for achieving GTR [6]. The first attempts to find techniques that ensure better intraoperative identification of brain tumor margins using fluorescent agents date back to before 1982 by Murray et al. [7], but it took nearly 30 years for the use of 5-ALA for FGR to be incorporated into daily clinical practice. These initial efforts laid the foundation for the advancement of fluorescence-guided resection in neurosurgery. Since the approval of 5-aminolevulinic acid (5-ALA) in 2017 (5-ALA; Gliolan^®^; photonamic GmbH and Co. KG [8]) for intraoperative guidance and better visualization of suspected high-grade gliomas, FGR has been shown to improve the volume of the tumor removed as well as the progression-free survival of patients [9]. In addition to 5-ALA, other fluorescent agents, such as sodium fluorescein (SF) [10] and indocyanine green (ICG) (Figure 1) [11] have also been utilized, while many researchers have turned their attention to the use of new dyes that will provide even better visualization of the tumor and will be capable of overcoming the limitations presented by these three fluorescent agents [6]

In this literature review, we will discuss the fluorescent dyes that are widely used in current clinical practice, as well as those that are still in experimental stages and show promising potential. We reviewed the relevant studies published on fluorescence-guided resection of GBM and evaluated the utility of each agent in terms of outcomes, GTR, and their sensitivity and specificity for the tumor. The database of Medline was searched, with the following search query: “(fluorescence-guided OR fluorescence dyes OR fluorescence agents) AND “glioblastoma” AND (surgery OR resection)” AND “Intraoperative”. We screened all retrieved studies by title and abstract according to inclusion criteria, which included the following: (1) the use of fluorescent substances intraoperatively to guide surgical resection, (2) surgical resection involving glioblastomas, and (3) studies focusing solely on fluorescence-guided surgery and not comparing it with other methods. Subsequently, the full texts of potentially eligible articles were evaluated based on the previous criteria, and, additionally, the following exclusion criteria were applied: (1) articles that were letters to the editor, and (2) language other than English.

## 2. 5-Aminolevulinic Acid (5-ALA)

5-ALA is the most widely used drug for the fluorescence-guided resection (FGR) of high-grade gliomas due to its metabolism into protoporphyrin IX (PpIX), a fluorescing agent. PpIX derived from 5-ALA remains the only fluorescent dye approved by the Food and Drug Administration (FDA) for the intraoperative visualization of high-grade glioma. Its approval underscores its established safety and efficacy in enhancing tumor delineation during surgery [8]. 5-ALA, which has no intrinsic fluorescence, is a naturally occurring intermediate molecule produced in the hemoglobin metabolic cycle. PpIX is a fluorescent dye that preferentially accumulated in the cells of gliomas, particularly in those of glioblastoma, and allows for tumor visualization with light of appropriate wavelengths [6,9]. The mechanism of action that leads to the selective accumulation of 5-ALA in GBMs and high grade gliomas cells depends on the following three factors: (1) the disruption of the blood–brain barrier exhibited by cancer cells, which allows 5-ALA to penetrate brain tissue, (2) the property of cancer cells to have increased metabolism of porphyrins and formation of PpIX due to the hyperactivity of 5-ALA synthase, and (3) the dysfunction of ferrochelatase (FECH) in tumor cells, an enzyme that converts PpIX into heme in normal brain cells [12].

The reduced expression of FECH in GBMs occurs through the decreased expression of its gene in the mRNA of glioblastoma cells, and it has been shown that this suppression is directly associated with the accumulation of PpIX [9,13]. Another enzyme whose expression is directly related to the fluorescence of PpIX in glioma cells is coproporphyrinogen oxidase (CPOX). CPOX is involved in the synthesis of heme, producing rotoporphyrinogen III (a direct precursor from PpIX) from coproporphyrinogen III through oxidative phosphorylation [14]. All these enzymatic changes, as well as the different characteristics of cancer cells, affect the production of heme and the citric acid cycle, and are directly involved in the mechanism of action and the fluorescence emitted by tumor cells after the administration of 5-ALA [12,13].

The FGR of high-grade gliomas using 5-ALA requires the presence of a surgical microscope connected to a light source (like the xenon light) in order to produce light with a wavelength of 370–440 nm, thus enabling the excitation of PpIX [14,15]. However, these microscopes require complete darkness, which can make it difficult to identify vascular structures, resulting in a higher likelihood of intraoperative bleeding. For this reason, today these microscopes are equipped with a blue filter so that the vessels and structures of the brain are easily visible during the darkness of tumor removal, thus avoiding intraoperative bleeding complications. Additionally, the use of a special filter is required that allows for the visualization of the tumor’s fluorescence with a maximum emission at 635–704, which corresponds to a bright red color [14,15]. The fluorescence derived from 5-ALA transformation depends on the density of the cancerous tissue, so at the center of the tumor, it appears dark red, while at its edges, it is light pink [9].

The administration of 5-ALA is performed orally 2–4 h before the surgery and at a dose of 20 milligrams per kilogram of body weight. After the administration of the medication, the patient has an increased risk of photosensitivity on the skin, which is why a 24 h stay in a dark place is required after administration to avoid this [14].

The first Phase III clinical study for 5-ALA was published in 2006 and included 322 participants who were equally and randomly assigned to 2 groups, with a group for FGR with the assistance of 5-ALA and another group for conventional microsurgical resection. The results showed that 5-ALA increased the successful removal of tumors in patients who received it by 29%, as well as their survival over the next 6 months without disease progression by 19.9% [16]. Since then, research in the field of FGR using 5-ALA has been extensive, and, according to Eatz et al., who performed a systematic review, 5-ALA has been shown to be capable of increasing the extent of resection of high-grade gliomas, prolonging the duration of patients without disease progression, as well as increasing the overall survival of patients [17]. According to a recent literature review by Mazurek et al., it is highlighted that the sensitivity and specificity rates for intraoperative identification of the margins of high-grade gliomas using 5-ALA are between 73.9–91.4% and 83.8–93.9%, respectively [18]. Additionally, 5-ALA appears to be able to assist equally effectively in cases of recurrence of high-grade gliomas, increasing the overall survival of patients, although it does not seem to be as effective for the progression-free survival period [19]. However, in certain cases of potential recurrence, La Rocca et al. report that the fluorescence derived from the transformation of 5-ALA in areas of radiation necrosis was incorrect, leading to false positive results with negative histological biopsy, which necessitates careful use in these areas [20].

According to a recent study by Kon et al., the use of image analysis tools, such as ImageJ (Version 1.54), can measure the brightness intensity of the PpIX fluorescence signal intraoperatively to differentiate between GBM, anaplastic astrocytomas, low-grade gliomas, inflammation, and necrosis. More specifically, they observed that GBMs have an average brightness of 134.5 arbitrary units (a.u.), while low-grade gliomas do not exhibit any detectable fluorescence, which may allow for easier differentiation [21].

Although the number of patients in this study is limited, the findings highlight a potential capability of 5-ALA in assisting with intraoperative localization of GBM and facilitating the rapid pathological diagnosis of tumors during surgery. This underscores its value as a tool for enhancing surgical precision and decision making in real time [21]. In addition to the utilization of 5-ALA in intraoperative guidance for the removal of high-grade gliomas, it has been successfully used for intraoperative photodynamic therapy (PDT) and for postoperative radiodynamic therapy of GBM, as these treatments increase patient survival without leading to major complications [22,23,24].

The single-arm INDYGO pilot trial that started in 2017 showed that 60% of patients who underwent intraoperative PDT and FGR with 5-ALA had 12 months of progression-free survival during their 12-month follow-up [22]. These results were further supported by the 3-year follow-up of the same patients, where it was observed that the median overall survival of the patients was 23.1 months, with 12-month survival observed in 80% of the patients. Moreover, after 5 years of intraoperative PDT use, it was observed that combining PDT with the optimal therapeutic regimen (maximum surgical resection, chemotherapy, and radiotherapy) offers an increase in the survival of patients with GBM without its use leading to major complications [23]. Similar encouraging results were reported by Schipmann et al. for a small group of patients with recurrent GBM. These studies, although they involve a limited number of patients, are quite encouraging and highlight the multiple potential uses of 5-ALA in the treatment of GBMs [25].

In recent years, research has explored the use of 5-ALA in radiodynamic therapy (RDT), a technique in which a radiosensitizing agent is administered and subsequently activated by ionizing radiation to selectively target and destroy cancer cells. During photodynamic therapy (PDT), PpIX is activated by light of an appropriate wavelength, leading to the production of reactive oxygen species (ROS), which induce the death of tumor cells. This approach holds promise for enhancing the effectiveness of radiation therapy in glioblastoma treatment [24]. The beneficial effects of 5-ALA as a radiosensitizing agent have been demonstrated both in vivo and in vitro, and the first Phase I/II clinical trial in humans was recently published [26,27]. This study involves patients with recurrent GBM who received 5-ALA prior to radiotherapy in order to evaluate whether it can enhance the destruction of neoplastic cells [27].

5-ALA has a high sensitivity and positive predictive value for high-grade gliomas; however, its specificity and negative predictive value are comparatively lower due to the signal attenuation at the tumor periphery [28]. Furthermore, the literature has documented cases of false positives associated with 5-ALA fluorescence, where fluorescent regions corresponded to demyelination areas in demyelinating diseases, such as multiple sclerosis, as well as inflammatory processes, infection sites (e.g., bacterial abscesses), non-glioma tumors, and regions of radiation necrosis and reactive gliosis in patients with recurrent GBM. These findings highlight the need for careful interpretation of 5-ALA fluorescence to avoid the misidentification of non-tumor tissue during surgery [20,29,30].

The limitations of its use include the inability to accurately detect periventricular tumors and areas of recurrent gliomas, where there is both false-positive fluorescence in regions altered postoperatively and by radiation, as well as false-negative fluorescence in areas that histopathologically correspond to tumor regions [30]. Additionally, its disadvantages include the high cost, the need for the patient to stay in a dark environment for 24 h after administration, the requirement for a specialized microscope, its time-dependent fluorescence capability and the autofluorescence in some areas [3,28,30]. Finally, apart from photosensitivity, its administration has been associated with side effects, such as transient hypotension and liver dysfunction [3,30].

## 3. Sodium Fluorescein (SF)

Sodium fluorescein (SF) is an FDA-approved fluorescent dye for ocular angiography. When administered intravenously, it binds to plasma proteins and selectively accumulates in high-grade gliomas, exploiting blood–brain barrier (BBB) disruption to enhance intraoperative tumor visualization [10,31]. Its use for the visualization of brain tumors was described as early as 1948, but the first Phase II trial (FLUOGLIO: Fluorescein-Guided Surgery for Resection of High-Grade Gliomas) to evaluate the safety of fluorescein-guided resection of high-grade gliomas began in 2011 [10,31,32]. The mechanism by which SF penetrates cancer cells differs from that of 5-ALA, as the disrupted blood–brain barrier allows SF to enter through vascular abnormalities at specific areas of the tumor, which then become visible due to the fluorescence of the substance [33]. SF fluoresces in the spectral band of 510–530 nm during surgery after being excited at 460–500 nm, just a few minutes after intravenous administration, allowing for the immediate intraoperative visualization of the target tumor [10]. Moreover, FS provides intraoperative visualization that closely resembles the tumor depiction seen in contrast-enhanced T1 images of preoperative MRI due to gadolinium accumulation. The dosage administered influences the type of microscope needed [10]. At low doses, a microscope with a special filter (for example the YELLOW 560 nm Filter, Carl Zeiss Meditec, Oberkochen, Germany) is required; whereas, at higher concentrations of FS, visualization is possible under any microscope with white light [10,31].

According to the results of the FLUOGLIO clinical trial, GTR was achieved in 82.6% of patients who underwent fluorescein-guided resection of suspected high-grade glioma tumors, without major adverse events or complications. Additionally, during the follow-up period, the median survival of the patients was found to be 12 months, with 56.6% and 15.2% of patients having progression-free survival at 6 and 12 months, respectively. The sensitivity of the substance for the identification of high-grade gliomas is 80.8%, and its specificity is 79.1%, which are quite close to those of 5-ALA [32]. Additionally, the meta-analysis by Smith et al. indicated that SF-guided resection can increase the GTR of these tumors by 29.5% compared to the control group, a percentage similar to that of 5-ALA [34]. The use of SF together with the YELLOW 560 nm filter has been suggested by some authors for the FGR of recurrent GBMs, as a study of 106 patients showed that GTR was achieved in 84% without any postoperative adverse effects [35].

SF is a very cost-effective fluorophore that can be used as an alternative to 5-ALA with equally good results. Compared to 5-ALA, it presents certain advantages, such as its use in emergency situations, as it can be administered intravenously at the start of the surgery and does not require the administration 2-4h before surgery like 5-ALA, as well as its low cost, its excretion by the kidneys, and the lack of a need for 24 h darkening as it does not cause photosensitivity in the skin. Nevertheless, due to its excretion by the kidneys within 24 h of administration, its use is not recommended in patients with end-stage renal failure [3]. Moreover, it is worth noting that at high doses (>20 mg/kg), cases of anaphylactic shock have been reported during its use in neurosurgical procedures [33,36]. One of the disadvantages of using SF is its non-selective entry into areas with a disrupted blood–brain barrier or in areas where it does not exist, such as the dura mater, as well as the risk of dye diffusion from the vessels into the surrounding tissue in cortical areas far from the target lesion [32]. For this reason, careful evaluation of the fluorescing tissues is required during its use in order to differentiate the pathological from the normal brain tissues [31].

Generally, its use is considered safe, although there has been no randomized clinical trial to confirm its safety. Recently, the combined use of SF with 5-ALA has been proposed, as it seems to lead to a beneficial outcome during the resection of GBMs by increasing the extent of resection (96% of the patients had GTR) and improving the prognosis [37].

## 4. Indocyanine Green (ICG) and Second Window ICG (SWIG) in Fluorescence-Guided Surgery

ICG is a near-infrared (NIR) fluorescent agent that has been used since 1972 for the examination of retinal and choroidal circulation in ophthalmology due to its ability to bind to plasma proteins and remain in the intravascular space [11,38]. It has also been utilized for angiography of the cardiovascular system, liver, and bile ducts, as well as in neurosurgery during the surgical treatment of aneurysms and arteriovenous malformations [30]. ICG has excitation at ~780 nm and emission ~800–850 nm. It is a small amphiphilic organic compound with the property of remaining within the blood vessels during intravenous administration. Its half-life is less than 200 s, and it is excreted by the biliary system. Due to this short half-life, administering it in small doses necessitates immediate imaging using an NIR imaging device [3,11].

The utilization of this fluorophore in imaging brain neoplasms is referred to as second window indocyanine green (SWIG) and requires the administration of ICG at a dose of 5 mg/kg, which is 10 times greater than the classical technique used for simple vascular imaging; it is administered 12–24 h before the surgery [3,11,30]. The mechanism of action of indocyanine green (ICG) in tumor visualization relies on blood–brain barrier (BBB) disruption and the enhanced permeability and retention (EPR) effect in neoplastic cells. In glioblastomas and other high-grade tumors, the endothelial integrity of neoplastic blood vessels is compromised, allowing ICG to extravasate into the surrounding tumor tissue and bind to albumin and other plasma proteins. This selective accumulation provides enhanced fluorescence contrast during imaging. In contrast, normal brain tissue maintains an intact BBB, preventing ICG from penetrating the parenchyma,. As a result, in non-tumor regions, ICG remains confined within the vasculature and is rapidly cleared from circulation, minimizing background fluorescence. This distinction between tumor and healthy tissue enhances intraoperative visualization, aiding in fluorescence-guided resection (FGR) and improving surgical precision [11,30]. Additionally, the SWIG leverages a phenomenon called the enhanced permeability and retention (EPR) effect to achieve ICG accumulation in the tumor. According to this phenomenon, in addition to the increased vascular permeability caused by endothelial disruption, ICG accumulation also occurs due to reduced lymphatic drainage and elevated levels of permeability mediators in tumor regions [11,39].

The primary distinction between conventional ICG angiography and SWIG lies in the dose and timing of ICG administration, which are optimized to harness the EPR effect and achieve superior tumor visualization. Furthermore, a specialized near-infrared (NIR) imaging system connected to the surgical microscope is required to detect the fluorescent tumor.

SWIG has been used in a variety of brain tumors, and its implementation in gliomas was first reported by Lee et al. in 2016 in 15 glioma patients [11,40]. According to the results of this report, SWIG demonstrated a sensitivity of 98% and a specificity of 45% for detecting high-grade tumors. Moreover, they mentioned that SWIG can reveal tumors at depths greater than 1 cm, and in eight of these patients, the tumor was already visible before the opening of the dura mater [41]. SWIG has also been utilized for imaging remaining tumors with increased angiogenesis after debulking, as well as for all intra-axial and extra-axial tumors, like meningiomas [3,11]. Finally, it has been proposed that SWIG could be used in stereotactic biopsies of intracranial tumors, providing immediate results about whether a tissue sample is part of the tumor based on its fluorescence [11].

The use of SWIG has the advantage of visualizing not only the tumor but also the surrounding tissue, making the tumor visible even during the incision of the dura mater [11]. However, compared to 5-ALA and fluorescein, it has lower sensitivity. Its disadvantages include the requirement of an NIR imaging device, the time-dependent nature of its fluorescence, the weakness of the dye signal, the associated cost, and the necessity for administration 24 h prior, which means that it cannot be used on an urgent basis. Furthermore, there are no randomized clinical studies for its safe use, nor data on its impact on GTR, progression-free survival, or overall survival [3,11,30,40]. However, it is worth mentioning that there is an ongoing Phase II/III clinical trial currently in the recruiting stage for the use of ICG in solid tumors, including gliomas (ID: NCT04723810), as well as a Phase I trial evaluating SWIG for all nervous system tumors, which is currently enrolling participants (ID: NCT05746104) [41,42]

## 5. Other Agents

Beyond these three fluorescent agents, the scientific community has focused on discovering new molecules that could be utilized in the FGR of gliomas. The aim is to overcome the limitations described earlier and achieve higher rates of GTR. Most of these substances are either in the preclinical stage or have been tested in a limited number of patients. They include peptide-based dyes, alkylphosphocholine (APC) analogs, monoclonal antibodies conjugated with NIR (near-infrared) dyes, and others [3,40].

### 5.1. Tozuleristide (BLZ-100)

**Figure 2:**
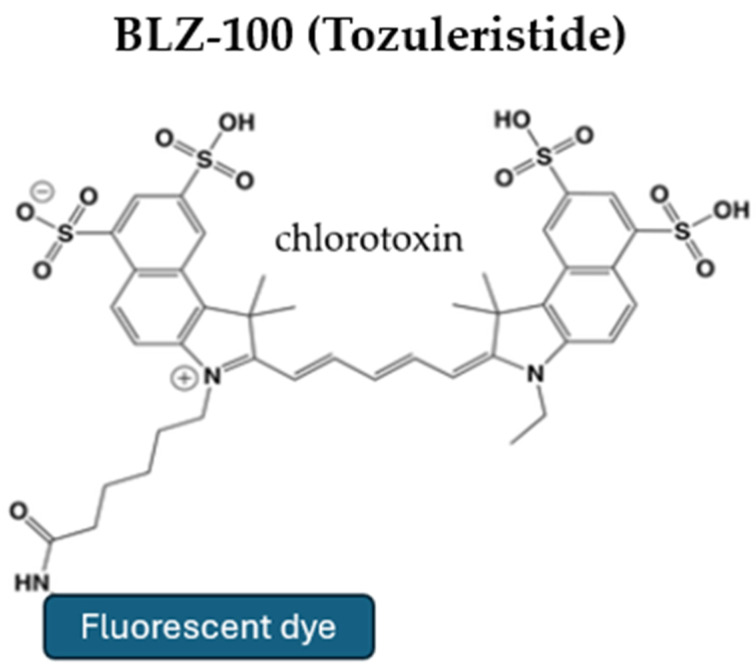
Structure of tozuleristide (BlZ-100). This tumor-targeting agent is derived from chlorotoxin, a component found in scorpion venom, and a fluorescent dye.

BLZ-100, also known as tozuleristide, is a chlorotoxin derived from scorpion venom, conjugated with ICG to fluoresce when used with NIR imaging devices (Figure 2) [3,30,43]. Both preclinical and Phase 1 clinical studies have shown that this compound is safe for human use and does not cause toxicity [44,45]. Its mechanism of entry into tumors is due to its molecular interaction with matrix metalloproteinases and calcium-dependent phospholipid-binding proteins, such as Annexin A2 [43]. BLZ-100 has been found to bind selectively to proteins and lipid rafts in gliomas, assisting in their fluorescence-guided resection (FGR) [46].

The first Phase 1 clinical study, published by Patil et al., demonstrated that the dye can be administered intravenously at doses ranging from 3 mg to 30 mg. At doses > 9 mg, the serum half-life is 30 min, while the fluorophore remains in the tumor for over 24 h. Additionally, it was observed that the concentration in high-grade gliomas was greater compared to low-grade gliomas, making it a promising tumor-specific dye candidate for FGR of GBMs [45]. Ex vivo analysis from this study revealed that BLZ-100 has a sensitivity of 82% and specificity of 89% for tumor localization and that it is also capable of increasing the surgical resection margins while minimizing damage to surrounding vital brain tissue [45]. However, the use of BLZ-100 requires administration at least three hours prior to surgery, making it unsuitable for emergency surgeries. Other disadvantages include the limited data on its safe use and efficacy. Finally, it is worth noting that a Phase I/II study on the use of tozuleristide for pediatric nervous system tumors was completed a few months ago, and the results are awaited [47].

### 5.2. Alkylphosphocholine (APC) Analogs

APC analogs are tumor-specific dyes, like BLZ-100, that appear to be a promising option for the fluorescence-guided resection (FGR) of gliomas [30]. Cancer cells in solid tumors have been shown to contain higher levels of phospholipid ethers compared to normal cells. More specifically, their cell membranes are enriched with cholesterol-rich lipid rafts, which facilitate the penetration and retention of APC analogs within cancer cells [46]. This property makes these analogs useful tumor-targeted dyes, which can be utilized both as positron emission tomography (PET) tracers for diagnostic imaging and as radiotherapeutic agents [48,49].

**Figure 3:**
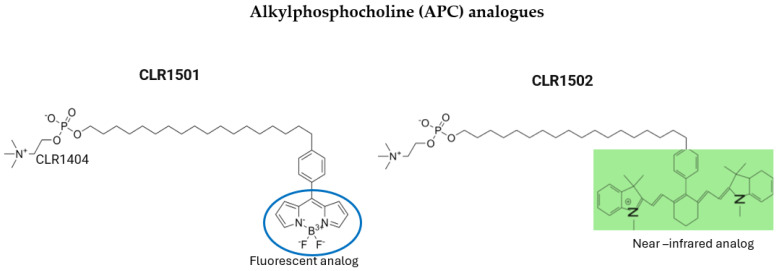
Structure of two commonly used alkylphosphocholine analogs. Both are based on the CLR1404 backbone, with the addition of a fluorescent analog in CLR1501 and a near-infrared analog in CLR150.

The effective use of APC analogs in the imaging of GBMs, demonstrated both in vivo and in vitro, led researchers to conjugate these substances with green (CLR1501) and near-infrared (CLR1502) dyes to achieve fluorescence-guided resection (FGR) in GBM murine models. CLR1502 and CLR1501, both derived from the conjugation of 18-(p-Iodophenyl)octadecyl phosphocholine (CLR-1401), are being investigated as potential therapeutic and diagnostic agents for the treatment of various cancers, including glioblastoma (Figure 3). The conjugation of CLR-1401 with fluorescent molecules enables targeted intraoperative imaging, as the compound selectively accumulates in cancer cells, allowing for improved tumor visualization during surgery [50,51].

Swanson et al. compared the effectiveness of CLR1501 and CLR1502 with 5-ALA, which is the standard dye used for FGR. They showed that CLR1501 has similar results to the standard dye for FGR, while GLR1502 appeared to have a better tumor uptake ratio compared to normal brain tissue, which is attributed to its potentially better penetration into the interior of cancer cells [51].

However, despite the encouraging results of this study, these agents face bioavailability challenges due to their high affinity for lipoproteins and albumin, which accelerates their clearance from circulation. Additionally, their clinical application is limited by the need for specialized microscopes with specific devices for tumor visualization and the lack of sufficient clinical data to fully establish their safety and efficacy in humans [3].

### 5.3. Tumor-Targeting Monoclonal Antibodies

Monoclonal antibodies conjugated with fluorescent agents, which have the property of selectively binding to cancer cells, have been utilized in the literature as potential alternative fluorophores in the FGR of brain tumors. Among them, the conjugated monoclonal antibodies targeting the Epidermal Growth Factor Receptor (EGFR), namely cetuximab–IRDye 800CW, panitumumab–IRDye 800CW, and ABY-029–IRDye 800CW, have garnered significant attention.

Cetuximab is a chimeric human–mouse antibody that selectively binds to EGFR, which is overexpressed or mutated in up to 70% of GBMs [30]. When conjugated with a fluorescent dye, cetuximab effectively identifies tumor cells both in vitro and in orthotopic animal models of GBM using a wide-field NIR imaging device [52]. Cetuximab–IRDye 800CW is typically administered intravenous two days before surgery to achieve optimal visualization of the tumor. The first Phase I clinical trial on a small group of patients demonstrated that FGR of GBM with Cetuximab–IRDye 800CW could enhance the extent of GTR without any adverse effects. Depending on the administered dose, the sensitivity and the specificity of this dye for targeting the tumor cells was 73.0–98.2% and 66.3–69.8%, respectively [53]. In 2023, a new Phase I clinical trial was started to identify the optimal dose of cetuximab–IRDye 800CW for FGR of high grade gliomas and to compare its effectiveness with the standard fluorescent dye, 5-ALA. The trial is currently ongoing and actively recruiting participants [54].

Panitumumab is a fully humanized antibody that also binds to EGFR, but it does not share the same epitope as cetuximab. Due to its nature, it appears to have a better safety profile, and, in conjunction with IRDye 800CW, it can be a promising tracer for FGR of GBM [55]. Napier et al. compared panitumumab–IRDye 800CW with 5-ALA in the FGR of GBM in patient-derived GBM xenograft models and evaluated the effectiveness of this new dye in determining the tumor margins and achieving better tumor resection. From the results of this study, it appeared that panitumumab–IRDye 800CW offers up to 30% higher tumor-to-normal tissue ratio uptake and over 10% higher accuracy in locating the center and margins of the tumor compared to 5-ALA [56] The infusion of panitumumab–IRDye 800CW must be administered 1–5 days prior to surgery, which means that, like cetuximab, it cannot be used in emergency surgeries based on the current data. In 2018, a phase I/II clinical trial was started to evaluate the potential side effects of the substance and to find the optimal dose for the FGR of GBM with panitumumab–IRDye 800CW. Currently patient recruitment is still ongoing and is estimated to be completed by the end of the year [57]. Moreover, another study was announced in 2024, which is set to evaluate the potential usefulness of panitumumab–IRDye 800CW in the intraoperative delineation of pediatric neoplasms; however, participant recruitment for this study has not yet begun [58].

ABY-029–IRDye 800CW is a synthetic anti-EGFR antibody conjugated with a fluorescent dye that is produced under pre-Good Manufacturing Practice conditions for tumor targeting and banding during surgical procedure [59]. The use of the synthetic antibody was proven to be safe in a Phase 0 study, as no toxicity was observed even at a dose 1000 times greater than the microdose required for tumor localization. From preclinical studies on FGR of mouse GBM, it appeared that there was up to 16 times greater fluorescence in the tumor areas compared to the normal ones, and that this fluorescence could be detected up to 48 h later [59,60]. In 2021, an early Phase I clinical trial was initiated to assess whether microdosing of ABY-029–IRDye 800CW produces detectable signals in tissues resected using fluorescence-guided resection (FGR) in patients with recurrent gliomas. The study has since been completed, and its results are currently awaited [61]. In all clinical trials, the administration of the dye was performed 1–3 h prior to surgery intravenously in a microdose, which offers an advantage compared to other EGFR antibodies that require administration 1–5 days before. The use of this synthetic molecule is still under investigation as there are not enough clinical studies, but it seems to be very promising. In addition to EGFR, which is overexpressed or mutated in GBM, glypican-1 is also overexpressed in these tumors and is associated with poor prognosis. Miltuximab is a chimeric antibody that targets the proteoglycan glypican-1 and when combined with IRDye-800CW it is a promising tracer for the FGR of GBM [62]. From preclinical studies in xenograft mouse GBM models, it appears that the antibody is capable of selectively accumulating in the tumor and causing strong fluorescence without causing significant side effects [63]. Currently, no clinical trial for its use in humans with GBM has been published; therefore, we await further studies to evaluate the potential application of miltuximab–IRDye-800CW in the GFR of GBM.

### 5.4. Tumor-Targeting Peptides and Protease-Activated Probes

Various tumor-specific targeting peptides had been used in the literature as potential molecular probes for better visualization of GBM. Among them, bombesin (BBN), a gastrin-releasing peptide receptor conjugated to the IRDye 800CW, as well as LUM015, a protease-activated fluorescent imaging agent, are the only ones that have progressed to clinical trials, with the second currently suspended.

BBN–IRDye 800CW has been studied in vitro and in vivo in an orthotopic glioma xenograft model as a dual-modality PET/near-infrared fluorescence imaging probe named ^68^Ga–IRDye800CW–BBN. This dye targets the gastrin-releasing receptor and is administered intravenously. Preclinical studies confirmed the safe use of the substance and led to the first-in-human prospective cohort study, which showed that the preoperative uptake of ^68^Ga–IRDye800CW–BBN by the tumor and the intraoperative fluorescence of the tumor have a perfect correlation [64] Additionally, the study showed that this dual-modality probe has a sensitivity and specificity of 93.9% and 100%, respectively, for tumor localization and that it does not cause serious adverse effects. In 2015, an early Phase I clinical study was initiated, which showed that the use of ^68^Ga–IRDye800CW–BBN in intraoperative GBM resection led to complete tumor removal in 82.76% of patients, while the median overall survival and progression-free survival were 23.1 and 14.1 months, respectively [65]. These results are quite encouraging, and more studies are expected for the further evaluation of its use. The ^68^Ga–IRDye800CW–BBN also has the advantage of being applicable in low-grade gliomas, unlike 5-ALA, and it can be utilized for both pre- and intraoperative targeted imaging through the same molecular receptor during LGG surgeries, according to the results of a phase I clinical trial. It is worth mentioning that due to the use of the radioisotope gallium-68, this dye is not suitable for patients with renal or liver failure [65,66].

LUM015 is a PEGylated protease-activated far-red fluorescent imaging probe, which can offer real-time imaging of cancer cells of various tumor types, including GBM [67]. LUM015 consists of a fluorophore and a fluorescence-quenching molecule which are linked together by a peptide backbone. In cancer cells, there is a group of proteases, cathepsins, which are highly upregulated in tumor cells and are capable of cleaving this peptide chain, thereby leading to the activation of LUM015 and, consequently, to the selective fluorescence of the tumor [68]. In 2019, a clinical trial was started to evaluate the optimized dose and the efficiency of the LUM015 imaging system in the visualization and the resection of primary and metastatic cancer in the brain. However, in 2022, it was suspended due to the need to modify the protocol before continuing the enrollment of patients, and it has not started again to this day [69]. The probe’s ability to activate LUM015 of cancer cells and the real-time imaging of the tumor mimics the FGR of GBM with 5-ALA, making it highly promising, provided there are sufficient data for its safe and effective use.

Following the same logic of activation upon entry into cancer cells, certain fluorescent probes have been developed that selectively bind to cyclic peptide chains of integrins or CD13, which are overexpressed in the neovasculature of tumors [3,70,71]. These peptides can be linked via a disulfide linkage to a fluorescent near-infrared dye, which remains inactive until it binds to the target peptide chain. When the peptide binds to the target cell, the disulfide linkage breaks, allowing the target to fluoresce. The Q-cRGD probe is a peptide that binds to the Arg-Gly-Asp (RGD) chain of integrin and has been shown in in vivo mouse glioma models to provide a tumor-to-background signal ratio of 2.56 after 3 h of intravenous administration, indicating its potential use in FGR of gliomas [71]. NGR and isoNGR contain the Asn-Gly-Arg and isoAsp-Gly-Arg chains, respectively, and bind to aminopeptidase N (APN), also known as CD13. These two are also being studied as new candidate fluorescent dyes for brain tumor surgery [71].

Urokinase-type Plasminogen Activator Receptor (uPAR) is overexpressed in solid cancers, including GBM. IRDye 800CW-AE344 is a uPAR-targeting peptide conjugated with the fluorescent substance IRDye 800CW which has been studied as a potential alternative optical probe for fluorescence-guided surgery. From the results of the preclinical study on orthotopic human patient-derived glioblastoma xenograft mouse models, it was shown that IRDye 800CW-AE344 is a promising fluorescent probe, as a tumor-to-background ratio above 4.5 was observed after its administration and it was shown to cross the blood–brain barrier in a 3D spheroid model. Furthermore, compared to 5-ALA, the in vivo fluorescence of IRDye 800CW-AE344 was greater. No serious side effects were reported, indicating a favorable safety profile [72].

Molecularly targeted protease-activated probes represent a promising tool for the fluorescence-guided surgery (FGS) of brain tumors. These probes are designed using substrates that contain a fluorescent dye linked to an inhibitor peptide, which remains inactive until enzymatic cleavage occurs. In the tumor microenvironment, proteases overexpressed in cancer cells but not in normal tissues selectively cleave the peptide, activating the fluorophore and enabling precise tumor visualization. One such probe, 6QC-ICG, is activated by tumor-associated cysteine cathepsins and incorporates indocyanine green (ICG) as the fluorophore, allowing for real-time intraoperative imaging of tumors. Beyond single-enzyme activation, dual-substrate probes have also been developed, requiring proteolysis by two different proteases, such as cathepsins and caspase-3, for activation. This dual-enzyme specificity enhances tumor selectivity and imaging accuracy, further improving the potential of protease-activated probes in brain tumor surgery.

Konecna et al. evaluated the potential for visualizing GBM in vitro and in vivo in orthotopic mouse models, using both the single-substrate probes 6QC-ICG and 6QC-Cy5 as well as the double-substrate probes AG2-FNIR and AG2-Cy5. 6QC-ICG and AG2-FNIR have a higher tumor to normal tissue ratio, but 6QC-ICG has a more intense fluorescent signal. Moreover, the ability of 6QC-ICG to selectively visualize GBM was confirmed in ex vivo human GBM biopsy material and a xenograft mouse model. The comparison of 6QC-ICG with 5-ALA showed a higher tumor-to-normal tissue fluorescent ratio and lower tissue autofluorescence, demonstrating the potential of using 6QC-ICG in the future as an alternative to 5-ALA [73].

In Table 1, we summarize the fluorescent dyes discussed in Section 2, Section 3, Section 4, and Section 5, along with their respective features.

## 6. Conclusions

GBM is the most aggressive primary brain tumor with low survival rates despite the implementation of optimal therapeutic approaches, which combine GTR, the administration of temozolomide, and radiotherapy. GTR is the cornerstone for increasing survival and progression-free survival rates. Fluorescence-guided surgery (FGS) has the potential to significantly enhance tumor visualization, aiding in more precise and complete resections, which is critical for improving patients’ outcomes. However, the fluorescent agents currently available in clinical practice face several limitations that hinder their effectiveness. One of the primary challenges is autofluorescence, where endogenous fluorophores in tissues generate background signals, reducing tumor-to-background contrast and making it more difficult to distinguish cancerous regions from healthy tissue. Additionally, many existing fluorescent dyes exhibit limited penetration depth, as their excitation and emission wavelengths fall within the visible spectrum, leading to signal loss in deeper tissues due to light scattering and absorption. To overcome these drawbacks, near-infrared (NIR) fluorophores are being explored, as they offer greater tissue penetration, reduced autofluorescence, and improved imaging contrast. Furthermore, the development of tumor-targeted fluorescent probes, activatable fluorophores, and dual-wavelength imaging systems aims to enhance specificity, minimize background noise, and provide real-time feedback for surgeons. Advancements in fluorescence imaging technology, including hyperspectral imaging and molecularly activated probes, hold promise for improving tumor delineation, maximizing resection, and ultimately extending patient survival. However, further clinical validation and optimization are necessary to integrate these next-generation fluorescent agents into routine surgical practice. Currently, a range of promising fluorescent agents is undergoing clinical and preclinical evaluation as potential alternatives to the standard fluorophore 5-ALA, aiming to overcome its limitations in fluorescence-guided resection (FGR) of glioblastoma (GBM). These novel fluorophores seek to address challenges, such as limited penetration depth, autofluorescence interference, and variability in tumor uptake, which can affect surgical precision. The successful completion of these studies could pave the way for the development of next-generation fluorophores with enhanced tumor specificity, deeper tissue penetration, and improved signal stability, ultimately contributing to more effective tumor resection. By refining intraoperative imaging and enabling more complete and precise tumor removal, these advancements have the potential to increase the survival rate and improve the overall prognosis for GBM patients.

## Figures and Tables

**Table 1:** Summary of the fluorophores currently used in glioblastoma fluorescence-guided surgery.

Agent	Administration	Advantages	Disadvantages	Limitations	Comments
5-ALA (PpIX)	Oral 3 h preoperation	Only FDA-approved dye for FGR of HGGs Enhance GTR and patient survival based on RCT	High cost Causes photosensitivity Limited in deep/periventricular tumors Cannot be used in LGGs It has been associated with side effects, such as transient hypotension and liver dysfunction	False positives with inflammation and necrosis Not ideal for recurrent or periventricular gliomas Needs a dark environment for 24 h	Can also be used in PTD and RTD therapy Sen.: 73.9–91.4% Sp.: 83.8–93.9%
Sodium fluorescein (SF)	IV, minutes before surgery	Cost effective Intravenous use at surgery star-useful for emergencies No skin photosensitivity Rapid visualization	Non-selective entry into areas with a disrupted BBB False positives in normal tissues (e.g., dura) Risk of anaphylaxis at high doses	Limited selectivity for tumor tissue SF may leak far from target Not recommended in end-stage renal failure (kidney excretion)	No RCT trial phase III to confirm its safety Can be combined with 5-ALA for improved GTR Sen.: 80.8% Sp.: 79.1%
Indocyanine green (ICG)	IV, 12–24 h preop	Visualizes tumors deeper than 1 cm Can show tumor before dura is opened Useful in various tumors Good for residual tumor detection	Requires NIR equipment Expensive Not usable in emergencies due to the need for 12–24 h preop administration	No large RCTs or long-term data Lower specificity (45%) No proven impact on GTR/survival yet—still in the clinical trial phase	ICG in brain tumors is referred as second window ICG (SWIG) Could be used in SBB Sen.: 98% Sp.: 45%
BLZ-100	IV administration at least three hours prior to surgery	Tumor-specific binding Safe for human use with no observed toxicity in clinical trials Selective accumulation in high-grade gliomas	Requires NIR imaging devices Not suitable for emergency surgeries Limited clinical data available	Efficacy in low-grade gliomas is not fully established Delayed administration time	In doses > 9 mg, the serum half-life is 30 min, while the fluorophore remains in the tumor for over 24 h
APC analog CLR 1501 CLR 1502	n/a	Tumor-specific targeting due to their interaction with lipid rafts Dual use: PET tracers and potential therapeutic agents Better tumor uptake and penetration compared to standard dyes, like 5-ALA	High affinity for lipoproteins accelerates clearance from circulation CLR1502 requires an NIR system, and both require specialized microscopes for tumor visualization No data about humans used for FGR	Bioavailability challenges Insufficient clinical data for safety and efficacy Specialized equipment needed	Only studies in human glioblastoma cells, glioblastoma stem-like cells, and rthotopic murine xenograf glioblastoma models
Cetuximab–IRDye 800CW	In early clinical trials IV 2 days before the surgery	Selectively targets EGFR, overexpressed in up to 70% of GBMs Proven safety and effectiveness in early clinical trials Enhances extent of GTR without adverse effects	Potential adverse effects based on dosage Limited to EGFR-expressing tumors	Ongoing Phase I trial to identify the optimal dose The sensitivity and specificity depend on the administration dose	Chimeric human–mouse antibody Phase I clinical trial to identify the optimal dose of cetuximab–IRDye 800CW for FGR is currently ongoing
Panitumumab–IRDye 800CW	In clinical trials IV 1–5 days before surgery	Better safety profile than cetuximab Up to 30% higher tumor-to-normal tissue uptake compared to 5-ALA Promising for FGR with improved accuracy in tumor margin determination	Still in the early stages of clinical trials Limited long-term data Limited to EGFR-expressing tumors	Requires further trials to establish optimal dosing Unsuitable for emergency surgery	Fully humanized antibody Phase I/II clinical trial for side effects and optimal dose for FGR in GBM still in recruitment status
ABY-029–IRDye 800CW	IV microdose 1–3 h prior to surgery in clinical trials	No toxicity observed even at high doses Safe in early clinical trials Promising for detecting tumor fluorescence 48 h after administration	Limited clinical data, particularly in humans Investigational phase with incomplete clinical results Limited to EGFR-expressing tumors	Needs further research for practical use	Synthetic antibody Preclinical studies show up to 16 times greater fluorescence in tumor areas compared to normal
^68^Ga–IRDye800CW–BBN	IV in clinical trial 1 h before the surgery	Dual-modality (PET and NIR fluorescence) imaging High sensitivity (93.9%) and specificity (100%) for tumor localization Applicable in both LGGs and HGGs Effective for pre- and intraoperative imaging	Requires specialized imaging devices (PET/near-infrared fluorescence imaging) Relatively limited by patient availability and specific imaging setups	Requires high-tech imaging equipment Not suitable for all tumor types Due to the used of radiotracers it is not suitable for patient with renal or liver failure	More studies are required
LUM015	n/a	Protease-activated, offering real-time tumor imaging Highly selective for cancer cells due to protease activation Promising for visualizing primary and metastatic tumors	Clinical trial suspended for protocol modifications Limited data on its full clinical efficacy	Requires further data on safety and effectiveness	No clinical trial for used in GBM FGR
Q-cRGD, NGR, isoNGR	n/a	Targets integrins (RGD) and aminopeptidase N (CD13) in tumors Shows potential for improved tumor-to-background ratio Potential for FGR of gliomas	Still early stages clinical trials. Not fully validated in human studies	Needs more clinical validation Primarily limited to preclinical data	No studies in humans.
IRDye 800CW-AE344 (uPAR-targeting)	n/a	Targets uPAR overexpressed in GBM Shows a high tumor-to-background ratio and favorable safety profile Crosses the blood–brain barrier in preclinical studies	Requires further investigation to confirm clinical applicability Limited human data	Needs more clinical trials to verify its full potential Limited research on its safety and long-term efficacy	More studies are required

5-ALA: 5-aminolevulinic acid, PpIX: protoporphyrin IX, FDA: Food and Drug Administration, HGGs: high-grade gliomas, LGGs: low-grade gliomas, RTD: radiodynamic, PTD: photodynamic, Sen.: sensitivity, Sp.: sensitivity, GTR: gross total resection, FGR: fluorescence-guided resection, SF: sodium fluorescein, ICG: indocyanine green, SWIG: second window indocyanine green, n/a: not applicable, PET: positron emission tomography, RCT: randomized clinical trial, BLZ-100: tozuleristide, IV: intravenous, SBBs: stereotactic brain biopsies, APC: alkylophocholine analog, BBB: blood–brain barrier, GBM: glioblastoma, NIR: near-infrared.
